# Detection of Type I and III collagen in porcine acellular matrix using HPLC–MS

**DOI:** 10.1093/rb/rbaa032

**Published:** 2020-10-01

**Authors:** Yang Zhang, Yi Chen, Bo Zhao, Jianping Gao, Leilei Xia, Fangyu Xing, Yingjun Kong, Yongchao Li, Guifeng Zhang

**Affiliations:** 1 State Key Laboratory of Biochemical Engineering, Institute of Process Engineering, CAS, Beijing 100190, China; 2 School of Life Science and Technology, Henan Institute of Science and Technology, Xinxiang, Henan 453003, China; 3 Beijing Biosis Healing Biological Technology Co., Ltd, Beijing 100026, China

**Keywords:** HPLC−MS, marker peptides, acellular matrix, collagen

## Abstract

Acellular matrix (ACM) has been widely used as a biomaterial. As the main component of ACM, collagen type and content show influence on the material properties. In this research, the collagen in ACM from different tissues of pig were determined by detection of marker peptides. The marker peptides of Type I and III collagen were identified from the digested collagen standards using ions trap mass spectrometry (LCQ). The relationship between the abundance of marker peptide and collagen concentration was established using triple quadrupole mass spectrometer (TSQ). The contents of Type I and III collagen in ACM from different tissues were determined. The method was further verified by hydroxyproline determination. The results showed that, the sum of Type I and III collagen contents in the ACM from small intestinal submucosa, dermis and Achilles tendon of pig were about 87.59, 81.41 and 61.13%, respectively, which were close to the total collagen contents in these tissues. The results proved that this method could quantitatively detect the collagen with different types in the ACM of various tissues.

## Introduction

Collagen is the main component of extracellular matrix (ECM), accounting for 25 − 30% of total protein. It is mainly found in animal skin, bone, tendon, ligament and other connective tissues [1–4]. Due to the unique biological characteristics, such as biodegradability, weak antigenicity and good biocompatibility, collagen becomes one of the most widely used biomaterials [5–7]. Collagen-based biomaterials include wound-healing materials, such as hydrogel, hemostatic sponge/powder, membrane and film, and tissue reconstruction materials [8–11]. However, collagen also shows some disadvantages such as low mechanical strength and rapid degradation. Thus, collagen is often used to composite with natural/synthetic polymers or inorganic materials to improve the material properties. Exogenous non-collagen materials often exhibit some uncontrollable properties, such as undegradability, acid release or other side effects [12, 13]. Acellular matrix (ACM) is a kind of novel biomaterial with ECM as raw material and removal of immunogenic substances such as cellular components and non-collagen extracellular proteins. ACM products on the market include VIDASIS™, Biodesign^®^ and Heal-All, etc. The main ingredients of ECM are collagen, glycosaminoglycans and low abundance proteins such as cytokines or growth factors [14, 15]. Currently, 27 types of collagen have been identified in mammals. Type I is the most abundant collagen in tissues, and Type III is often associated with Type I. The ratios of collagen Type I to III in different tissues are not same and change with age [16–21]. Thus, the ACM obtained from various tissues show different characteristics such as biodegradability, cell adhesion or tissue induction [22]. So, detection of collagen with different types in ACM is necessary. 

Many methods for collagen detection have been reported. The ultraviolet spectrometry and histochemical staining were usually used to identify different types of collagen. The quantitative detection methods included ultraviolet spectrophotometry, hydroxyproline (Hyp)-based method, HPLC, Sirius red staining and immunoassay [23]. At present, Hyp-based method was the most commonly used to detect collagen content, but the proportions of Hyp in collagen from various sources or tissues were different. The result was affected by the degree of hydroxyproline hydrolysis [24]. The principle of immunoassay method was antigen−antibody combination. This method was sensitive and specific, but it was not suitable for tissue collagen detection, since each step required cleaning and it showed poor reproducibility for different materials [25]. The Sirius red staining method was sensitive, but it was affected by the thickness and position of the tissue section, so it was not suitable for non-uniform tissue [26]. ACM is not soluble in aqueous solution, and spectrophotometry or staining-based methods are not suitable for the detection of tissue collagen. Immunoassay-based method is only applicable for soluble sample. Hyp-based method is not suitable for quantitative detection of collagen with different types.

In this study, a marker peptide-based method was employed with detection of collagen in ACM. Type I and III collagen standards were digested with trypsin and detected by ions trap mass spectrometry (LCQ) to confirm the marker peptides. The marker peptides were detected by triple quadrupole mass spectrometer (TSQ) to plot the standard curves, and the contents of Type I and III collagen in ACM were calculated according to the standard curves. The Hyp was detected by HPLC to calculate the total collagen content in ACM to verify the above results. The method that used marker peptides would not be affected by other proteins, and had a high precision, so it could be used to identify the collagen types and accurately detect the collagen contents in ACM.

## Materials and methods

### Materials and instruments

Fresh small intestinal submucosa (SIS), Achilles tendon and dermis of pig (generously gift from Beijing Biosis Healing Biological Technology Co., Ltd), Sequence-grade trypsin (Promega, USA), Type I and III collagen standards (YO Proteins AB), DNA extraction kit (Beijing Noble Technology Co., Ltd) and DNA quantitative kit (Sigma, USA).

Spectra Max M5 multi-function microplate reader (Molecular Devices, USA), HC-2518R high-speed refrigerated centrifuge (Anhui Zhongke Zhongjia Scientific Instrument Co., Ltd). LCQ and TSQ (Thermo Fisher Scientific, USA), and analyzed by Xcalibur 3.1 software.

### Enzymatic hydrolysis of collagen standard

The collagen standards were digested by trypsin. A 10-mg sample (collagen standard) was added to 10 ml ammonium bicarbonate buffer (50 mM, pH 8.0). The collagen was denatured by heating at 100°C for 10 min, and then cooled to room temperature. A 200-μl trypsin buffer (1 mg/ml, 50 mM ammonium bicarbonate, pH 8.0) was added into the collagen solution. Collagen was digested at 37°C for 16 h. The digested mixture was detected by High performance liquid chromatography–mass spectrometry (HPLC−MS).

### Preparation of ACM from different tissues of pig

The ACM from various tissues of pig were prepared as follows. The SIS, Achilles tendon and dermis of pig were cut into 5 cm × 5 cm or 10 cm × 10 cm slices, and then repeatedly frozen and thawed between 37°C and −80°C for 30 min. The tissues were treated with 0.1 M NaOH for 2 h. 0.03% trypsin solution (phosphate buffer, pH 7.4) was added to treat for 30 min. The samples were washed with deionized water for 30 min after each step.

### Detection of the Type I and III collagen

The on-line chromatographic separation was performed on an Agilent Zorbax SB C_18_ column (150 × 2.1 mm, 5 μm). The mobile phase consisted of water with 0.1% formic acid (A) and 60% acetonitrile with 0.1% formic acid (B). A gradient elution (0–120 min, 5–100% B) was introduced at a flow rate of 0.2 ml/min at 30°C. The injection volume was 50 μl.

Mass spectrometry conditions: the spray voltage was 4.5 kV, capillary temperature was 300°C, MS scan range was *m*/*z* 300 − 2000. The zoom scan and tandem MS (MS/MS) functions were performed in data-dependent mode. The collision energy value was set at 35%. The monitored target ion of Type I collagen was *m*/*z* 773.9, and Type III collagen was *m*/*z* 533.09.

### Detection of DNA residues

The cell residues in samples were characterized by DNA contents. The samples (porcine tissue) were lyophilized after each step in the decellularization process. The dry samples were used to extract DNA, and then detected by DNA quantitative kit.

### Total collagen detection

The contents of Hyp and collagen from various tissues were detected as reported [27–29].

### Statistics analysis

One hundred microliter collagen standard digestion was accurately detected using HPLC−MS. This step was repeated three times to verify the precision of HPLC−MS method. The digestion was stored at −20°C and, respectively, detected at 1, 10 and 15 days to verify the stability.

## Results

### Identification of marker peptides in the digested Type I and III collagen

**Figure 1:**
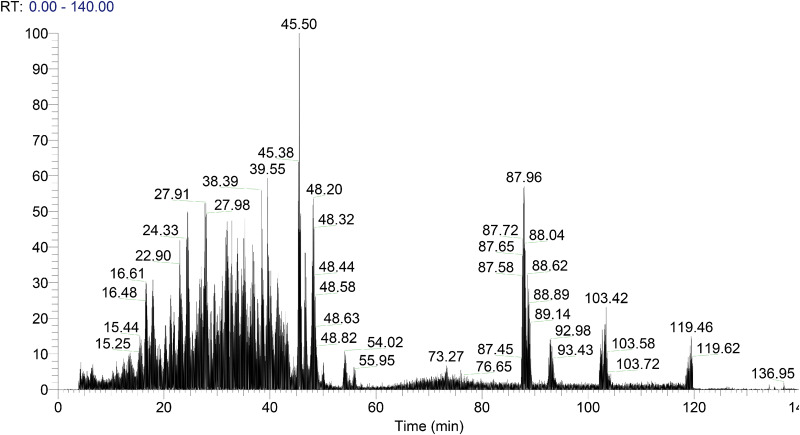
Total ions mass spectrum of porcine collagen digestion.

The porcine collagen standards were digested with trypsin, and detected by LCQ. Figure 1 shows the total ions mass spectrum of the collagen peptides.

Collagen was digested with trypsin to result amounts of low molecular weight peptides. The mass spectrometric information of collagen peptides was searched by the BLAST multi-sequence alignment and Bioworks software. The peptides were input into the protein information database, and cannot be found in other ECM proteins. The peptides detected only in the porcine Type I collagen were Type I collagen marker peptides. Similarly, the Type III collagen marker peptides were detected only in Type III collagen. The following SEQUEST criteria was used as peptide filter, Xcorr≥1.5 for +1 charged peptide, Xcorr≥2.0 for +2 charged peptide, Xcorr ≥ 2.5 for +3 charged peptide, and Deltacn > 0.1. The eligible peptides were considered to be the positive results. The peptide, which its ion chromatogram could be extracted from the mass spectrum of low concentration sample and the abundance was high, in addition its signal-to-noise ratio S/*N* > 10, could be confirmed as the candidate marker peptide. The candidate peptides with less amino acids, fewer hydroxylation sites, and higher matching rate of secondary fragment ions, were more suitable to be selected as the marker peptides.

**Figure 2:**
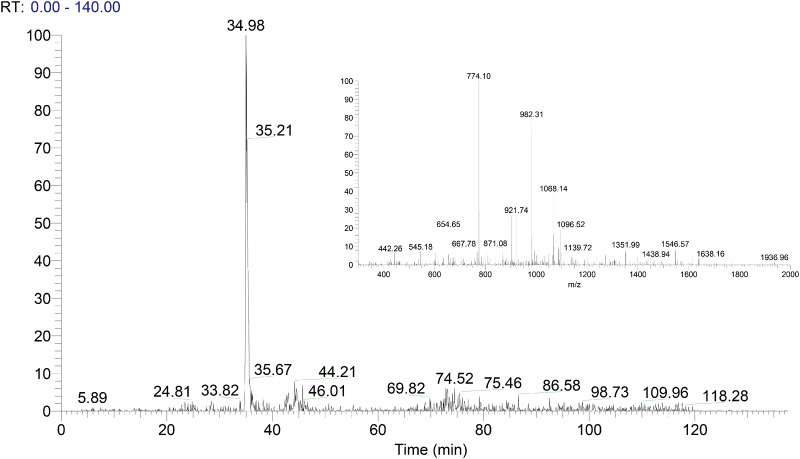
Mass spectrum and zoomscan spectrum (inset) of the ion, *m*/*z* 773.9 detected in the digested porcine Type I collagen.

**Figure 3:**
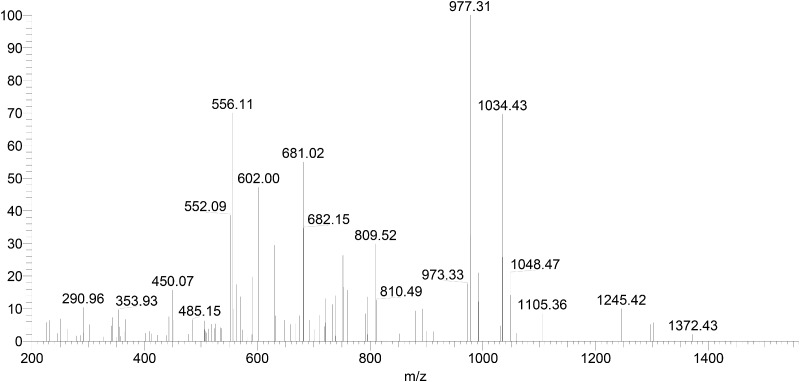
MS/MS Spectrum of the porcine Type I collagen marker peptide.

The acquired MS/MS data were searched against the collagen database. The candidate marker peptides of Type I and III collagen are listed in Tables 1 and 2. Figures 2 and 3 are Mass spectrum, zoomscan spectrum (inset) and the MS/MS spectrum of the peptide GETGPAGPAGPVGPVGAR, respectively. The MS/MS spectrum highly matched with the theoretical value from the database, and its abundance was high. Therefore, the peptide GETGPAGPAGPVGPVGAR was confirmed as the marker peptide of type I collagen. Similarly, the peptide GPPGAVGPSGPR was identified as the type III collagen marker peptide.

**Table 1:** The candidate marker peptides of Type I collagen from pig

No.	Polypeptide segments	MH+	Z	XC	DeltaCn
P1	R.GPP*GESGAAGPAGPIGSR.G	1551.64	2	4.016	0
P2	R.GETGPAGPAGPVGPVGAR.G	1546.8	2	3.638	0.945
P3	R.GPP*GPMGPPGLAGPP*GESGR.G	1818.01	2	3.438	0.072
P4	R.VGPP*GPSGNAGP*PGPP*GPAGK.G	1813.95	2	3.387	0
P5	K.DGEAGAQGPPGPAGPAGER.G	1691.74	2	3.347	0.876

Note: ‘P*’ represents hydroxyproline.

**Table 2:** The Candidate marker peptides of Type III collagen from pig

No.	Polypeptide segments	MH+	Z	XC	DeltaCn
P1	R.GPPGP*QGLP*GLAGAAGEP* GR.D	1804.94	2	3.226	0.15
P2	K.DGPP*GPP*GSSGAP* GSPGVSGP*K.G	1923.98	2	2.812	0
P3	R.GP*PGAVGPSGPR.G	1065.17	2	2.541	0.097
P4	R.GPPGP*P*GTNGAP*GQR.G	1408.46	2	2.482	0.005
P5	R.GETGPAGPAGAPGP*AGSR.G	1523.59	2	2.243	0.145

Note: ‘P*’ represents hydroxyproline.

### Standard curves of the marker peptides

**Figure 4:**
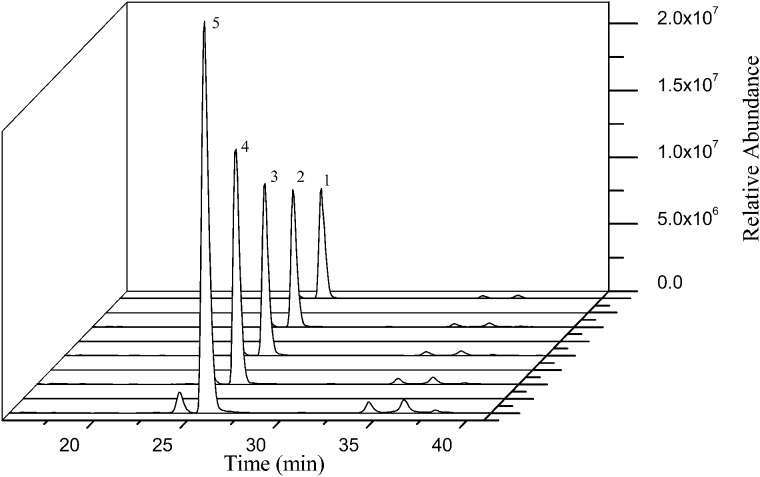
Mass spectrum of different concentrations of Type I collagen marker peptide GETGPAGPAGPVGPVGAR.

The collagen standard digestions were sequentially injected from low to high concentrations. According to the conditions of quantitative detection, Type I collagen marker peptide monitored target ion was *m*/*z* 773.90, and Type III was *m*/*z* 533.09. Figure 4 is the mass spectra of different concentrations of Type I collagen marker peptide. The linear regression curves that between the peak areas (×10^7^) and concentrations of Type I and III collagen were *y* = 17.13*x* + 0.41 (*R*^2^ = 0.9985) and *y* = 4.15*x* + 0.16 (*R*^2^ = 0.9981), respectively.

### Preparation and characterization of ACM

Hyp was a marker amino acid in collagen, which was not present in other proteins except for a small amount of elastin and complement. The proportion of Hyp in mammalian collagen was about 12% [14, 30, 31]. Hyp was detected by HPLC to calculate the content of collagen in ACM.

**Figure 5:**
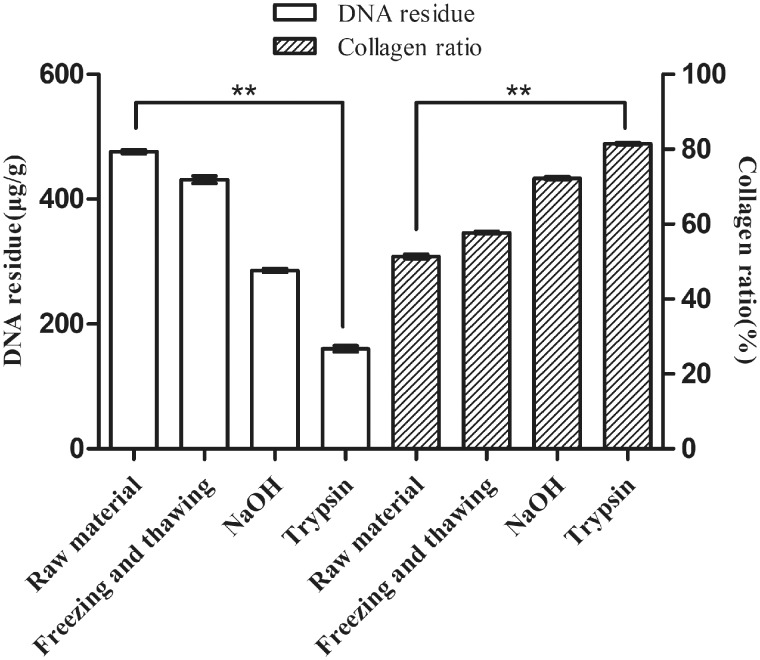
Changes in collagen ratio and DNA residue after each step of the decellularization process.

The physical, chemical and biological treatments were used to prepare the ACM in this study [32]. The contents of Hyp in tissues such as SIS were detected using HPLC with the decellularization process. As shown in Fig. 5, the proportions of collagen in SIS increased with the decellularization process and the DNA residues decreased with it. The content of collagen in fresh SIS was 51.42% or less, and that of homemade ACM of SIS was ∼81.36%. The DNA residue was ∼165.35 μg/g after treating with trypsin. In the decellularization process, freezing and thawing could remove some cells, but the effect was not significant. A certain concentration of NaOH could destroy the cells through strong alkali. NaOH was also used for degreasing by the saponification reaction, where grease was dissolved in water in the form of glycerin and soap. The trypsin could further dissociate the cells by enzymatic hydrolysis of proteins on the cell surface [33, 34].

The collagen content in ECM was more than 80%. According to the results, the degree of decellularization and the purity of collagen in ACM were high. The prepared ACM material could be used to detect the content of Type I and III collagen.

### Contents of Type I and III collagen in ACM

A 10-mg ACM from tissue of pig was weighed and digested with trypsin. The digested mixture was detected by HPLC−MS. The contents of Type I and III collagen were calculated according to the standard curves.

**Figure 6:**
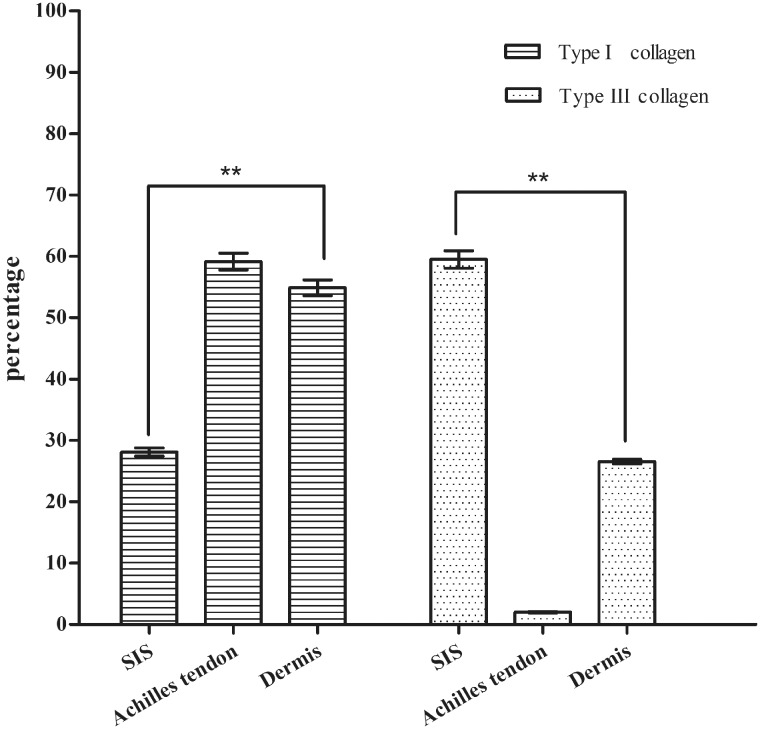
The contents of Type I and III collagen in ACM from different tissues of pig.

As shown in Fig. 6, the contents of Type I collagen in the ACM of porcine SIS, dermis and Achilles tendon are 28.09 (±0.67)%, 54.87 (±1.31)% and 59.13 (±1.38)%, respectively. Those of Type III collagen are 59.5 (±1.42)%, 26.54 (±0.35)% and <2%, respectively.

### Precision and stability of the HPLC−MS method

This study verified the linear relationship, precision and stability of the HPLC−MS method. The standard curves of porcine Type I and III collagen showed the good linear relationships. The peak areas of the extracted ion were brought into the formula of relative standard deviation (RSD), respectively. The RSD of method precision and stability was 2.38 and 7.1%, respectively. The results indicated that the HPLC−MS method had a high precision, and it was stable within 2 weeks.

## Discussion

Type I and III are the most common collagen in ECM. The contents of Type I and III collagen were detected using HPLC−MS. The total content of Type I and III collagen was close to the total collagen content that obtained by Hyp-based method. The results indicated that the HPLC−MS method could be used to detect the accurate content of collagen in ACM. It was found that the content of Type III collagen was higher than Type I in SIS. There were many crisscross blood vessels in the porcine SIS, and Type III collagen was mainly present in the ECM of blood vessel, lymphatic vessel, intestine and stomach, so the results were reasonable. The content of Type III collagen was much lower than that of Type I in Achilles tendon. According to the report [35], collagen was ∼70% of the dry Achilles tendon, and Type I was 95% in Achilles tendon collagen. The results obtained by HPLC−MS-based detection corresponded to the report by Waterston. The content of Type I collagen was higher than Type III in dermis of pig. Karayi found the content of Type I collagen was slightly higher than Type III in normal skin by the immunohistochemistry method [36]. The HPLC−MS method was reliable. 

This method showed great application potential in the field of medicine and material. For Masson’s trichrome staining, western blotting and other methods, the arrangement and density of collagen fibers and dyed area were used to detect collagen in the process of wound repair, but these methods were often unstable [37]. The HPLC−MS method could accurately detect the types and contents of collagen *in situ*. In addition, the collagen type and ratio in tissue were certain, so the change of the ratio might indicate the occurrence of some diseases, such as pulmonary fibrosis, arteriosclerosis and aneurysm, etc. The HPLC−MS method could be used to investigate the relationship between diseases and collagen change [38–40]. The degradation of implanted-collagen *in vivo* was usually detected by the radioactive labeling or Sirius red method, etc. The HPLC−MS method could detect the residue of collagen-based biomaterial by detection of marker peptides, avoiding the influence from the tissue collagen [41, 42]. The HPLC−MS method could also be used to develop the composite materials by cross-linking the collagen with other materials [12, 13].

## Conclusion

A collagen detection method was established based on HPLC−MS and marker peptides, which could detect the contents of Type I and III collagen in tissue. It was found that the sum of the Type I and III collagen contents was close to the total collagen content, and the contents of Type I and III collagen in different tissues were consistent with the distribution that collagen in the body. The method established in this work exhibited satisfied precision and stability, which would not be disturbed by other proteins such as fibronectin, laminin, etc.

## Funding

This work was supported by the National Key Technology R&D Programs of China (2018YFA0108200 and 2018YFC1106400), Beijing Nova Program (Z181100006218013, XX2018008) from the Beijing Municipal Science and Technology Commission, Key scientific research project plan of colleges and universities in Henan Province (17A530002) and the Guangzhou People’s Livelihood Science and Technology Project of China (201803010086). 

*Conflict of interest statement*. None declared. 
